# Electrochemical Degradation of Perfluoroalkyl Sulfonates via Sulfonate to Carboxylate Conversion

**DOI:** 10.1002/anie.202525896

**Published:** 2026-01-09

**Authors:** Stella A. Fors, Richard J. Monsky, Emily R. Mahoney, Christian A. Malapit, William R. Dichtel

**Affiliations:** ^1^ Department of Chemistry Northwestern University 2145N Sheridan Rd Evanston IL 60208 USA

**Keywords:** Degradation, Desulfonation, Electrochemistry, Organofluorines, PFAS

## Abstract

Efficient, scalable, and well‐understood methods for degrading per‐ and polyfluoroalkyl substances (PFAS) are essential for limiting their numerous negative human health and environmental effects. Electrochemical methods are promising for PFAS degradation but are currently not yet well developed for use in non‐aqueous conditions relevant for PFAS sorbent regeneration without resorting to specialized electrode materials. Herein, we report the mediated electrochemical conversion of perfluoroalkyl sulfonates to carboxylates using commercial Pt electrodes in acetonitrile. Perfluorooctane sulfonate (PFOS) was converted primarily to perfluorooctanoic acid (PFOA) alongside several shorter‐chain carboxylates through a proposed radical desulfonation and hydroxide coupling process explored in a detailed mechanistic study. Following the near‐complete conversion of PFOS to perfluoroalkyl carboxylates, all species are mineralized to fluoride and non‐fluorinated carbon byproducts using established low‐temperature DMSO/NaOH conditions. HPLC‐MS, ion chromatography, and quantitative nuclear magnetic resonance (NMR) methods determined a significant loss in fluorine and carbon balance after electrochemistry, which we attribute to the production of volatile byproducts. This degradation approach provides new insights into PFAS degradation mechanisms under highly oxidative, non‐aqueous conditions and highlights the potential for organic electrochemistry to address environmental challenges by promoting controlled and selective destruction pathways for common organic pollutants.

## Graphical abstract

We demonstrate a novel approach for the degradation of perfluoroalkyl sulfonic acids (PFSAs) involving a controlled, electromediated, sulfonate‐to‐carboxylate headgroup conversion. We apply this method to shorter chain PFSAs and provide key mechanistic insights that contribute to a broader understanding of the molecular pathways underlying pollutant degradation and highlight the power of organic electrochemistry in addressing environmental challenges.

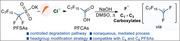

## Introduction

Per‐ and polyfluoroalkyl substances (PFAS) are a class of synthetic chemicals that feature multiple C–F bonds and a polar head group (e.g., carboxylate or sulfonate ions) that found extensive use in commercial and industrial applications for their thermal stability and omniphobic properties.^[^ ^1^ ^]^ However, their environmental persistence and bioaccumulation have been linked to severe health risks, including cancer and immune dysfunction, prompting global regulatory restrictions, and urgent demands for effective remediation strategies.^[^ ^1^ ^]^ Several degradation approaches, including thermal,^[^ ^2^, ^3^ ^]^ photochemical,^[^ ^4^ ^]^ sonochemical,^[^ ^5^ ^]^ and electrochemical methods,^[^ ^6^ ^]^ have been developed to mineralize PFAS to fluoride and benign byproducts. Among these, electrochemical degradation methods are attractive for their potential scalability, cost‐effectiveness, and tunable electron transfer pathways.^[^ ^7^ ^]^ Currently, electrochemical methods for PFAS degradation have been limited to using specialized metal oxide or boron‐doped diamond electrodes in aqueous conditions, and feature limited mechanistic insight into the destruction process, or invoke uncontrolled mechanisms primarily driven by hydroxyl radical species.^[^ ^8^, ^9^, ^10^, ^11^ ^]^ The poor understanding of the reactivity of PFAS and their intermediate degradation products under these reaction conditions detracts from our ability to rationally develop, evaluate, and optimize degradation technologies.

**Figure 1:**
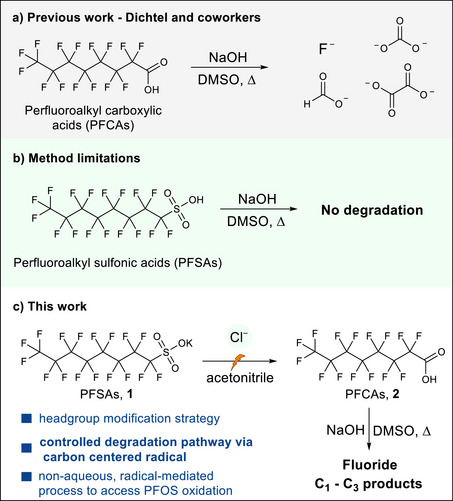
Electrochemical degradation of perfluoroalkyl sulfonates via headgroup modification to carboxylates. a) Previously reported work demonstrated the base‐mediated mineralization of perfluoroalkyl carboxylic acids (PFCAs) to fluoride and non‐fluorinated carbon byproducts^[^ ^2^, ^15^ ^]^; b) Perfluoroalkyl sulfonates are resistant to degradation under the same conditions; c) Perfluoroalkyl sulfonate degradation by an initial electrochemical conversion to PFCAs followed by subsequent base‐mediated mineralization.

Having developed novel PFAS sorbents that are regenerable with non‐aqueous solvents,^[^ ^12^, ^13^, ^14^ ^]^ we are exploring PFAS reactivity in such environments with a long‐term goal of developing mineralization procedures that operate in these regeneration liquids. Non‐aqueous solvents support broad potential windows for electrochemistry, including highly positive oxidation potentials previously used for the destruction of perfluoroalkyl sulfonates.^[^ ^8^ ^]^ The reactivity of these sulfonates is also of interest since other classes of PFAS degrade readily under the energetically mild thermochemical conditions reported by Dichtel and coworkers (Figure 1a).^[^ ^2^ ^]^ Sulfonates resist degradation under these conditions (Figure 1b), but we reasoned that electrolysis might only need to convert the sulfonate to a carboxylate or other reactive group, such that complete defluorination via electrochemistry would not be required. We hypothesized that the generation of carbon‐centered radicals from perfluorooctane sulfonate (PFOS, **1**) would enable the subsequent formation of perfluorooctanoic acid (PFOA, **2**) after engaging with an OH radical (Figure 1c). Herein, we report an electrooxidative, mediated conversion of perfluoroalkyl sulfonates to carboxylic acids, enabling mineralization in a second, thermochemical step. We undertook a detailed mechanistic study for the head group conversion and demonstrate compatibility with 8, 6, and 4‐carbon chain perfluoroalkyl sulfonic acids (PFSAs). ^19^F and ^13^C nuclear magnetic resonance (NMR) spectroscopy, high‐performance liquid chromatography‐mass spectrometry (HPLC‐MS), and ion chromatography were employed to characterize and quantify the reaction products, revealing significant losses in carbon and fluorine balance, which we attribute to the formation of volatile products. Nevertheless, this work unveils the electrochemical reactivity of perfluoroalkyl sulfonates in non‐aqueous solvents using inexpensive, commercially available electrode materials to access much less thermally stable and more easily degradable perfluoroalkyl carboxylates. This report demonstrates the potential of organic electrochemistry to address environmental challenges by enabling selective and controlled degradation pathways of organic pollutants and promises further advancements in green chemistry and PFAS remediation.

## Results and Discussion

Perfluorooctane sulfonate (PFOS) is transformed to perfluorooctanoic acid (PFOA) and shorter chain PFCAs when oxidized in an electrochemical cell using Pt electrodes in acetonitrile (MeCN). The reaction was performed in an undivided cell using an IKA ElectraSyn 2.0 instrument, which was used to optimize reaction conditions by varying the solvent, supporting electrolyte, electrode material, and amount of charge passed. PFOS degradation is solvent‐dependent, as effective desulfonation was only observed in acetone and MeCN. Solutions of other solvents, including dimethyl sulfoxide (DMSO), sulfolane, or dimethylacetamide, resulted in little to no (<5%) conversion (Figures S9 and S10). Although early experiments performed in acetone suggested that partial desulfonation was occurring, these findings were difficult to reproduce, and an intractable mixture of acetone‐derived byproducts further complicated the analysis. In contrast, optimized conditions with acetonitrile under constant current electrolysis achieved PFOS conversion of >99% to form a mixture of PFCAs containing between four and eight carbons. After transitioning to MeCN as the solvent, we screened various electrolytes to improve reaction efficiency and increase solvent conductivity. We found that sodium chloride (NaCl) provided the greatest conversion of the starting material. Despite initial poor solubility in MeCN, the NaCl dissolved as the reaction proceeded, suggesting its participation in the solution phase. Furthermore, when the reaction was performed under a constant potential regime with and without NaCl, the standard reaction conditions including NaCl sustained a greater current than the conditions without NaCl. This difference indicates NaCl is soluble enough to act as an electrolyte and that the reaction generates charged species that increase the conductivity of the reaction medium (Figure S8). Reticulated vitreous carbon (RVC) was initially chosen as the electrode material, as it produced 90% conversion of PFOS to various PFCAs after the passage of 25 Faradays/mole of substrate (F mol^−1^). However, Pt plates provided a near‐quantitative conversion of PFOS after 10 F mol^−1^ passed with fewer fluorinated byproducts than RVC, as determined by ^19^F NMR spectroscopy, indicating a more efficient desulfonation reaction. The inferior performance of RVC may arise from carbon‐centered radicals grafting to the electrode surface, which has been described in other reactions.^[^ ^16^ ^]^

**Figure 2:**
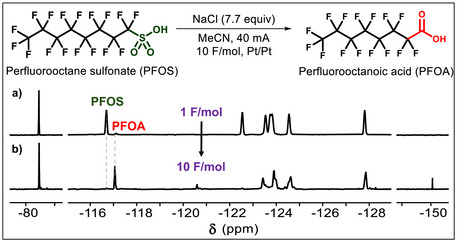
Electrochemical conversion of PFOS to PFOA. Standard reaction conditions and ^19^F NMR spectroscopy performed after PFOS electrooxidation, where either 1 F mol^−1^ a) or 10 F mol^−1^ b) are passed (DMSO‐*d*_6_, 298 K, 600 MHz). The signals corresponding to the α ‐CF_2_ fluorines used to identify PFOS (–116.71 ppm) and PFOA (–117.21 ppm) are labelled. Chemical shifts are referenced to α,α,α‐trifluorotoluene at –63.2 ppm.

The electrochemical conversion of PFOS to PFCAs was characterized using ^19^F NMR spectroscopy, and the PFOS conversion and product distribution as a function of charge passed in the electrochemical experiment were quantified using HPLC‐MS. Given its high sensitivity and low background signal, ^19^F NMR spectroscopy is useful for monitoring the degradation reaction and identifying transient intermediate products. Several of these fluorine‐containing groups, such as the ─CF_3_ and the ─CF_2_─ groups adjacent (α) to the head group, have characteristic chemical shift ranges that can be used to quantify each unique PFAS species observed. Additionally, using an internal standard and extended relaxation delay (D_1_) time allows for the quantification of the α CF_2_ signal in PFOS (–116.71 ppm), which was used as the diagnostic peak to measure its concentration in solution as a function of charge passed. Using this approach, we found the minimal amount of charge for complete PFOS conversion was 10 F mol^−1^ (Figure 2). As the reaction progresses, the concentration of byproducts with resonance signals around –82.2 ppm increases as the concentration of PFOS decreases until 10 F mol^−1^ of charge is passed, at which point > 99% of PFOS has reacted. In addition to the production of PFCAs, a small amount of tetrafluoroborate anion (BF_4_^−^) is observed at –151 ppm, likely corresponding to a reaction between HF and borosilicate glass during the electrolysis reaction (Figures S2, S17, and S18).

The degradation of PFOS under these conditions appears to produce volatile fluorine‐containing byproducts. Integrating all signals in the CF_3_ region reveals that all detected PFAS accounts for 50% of the starting amount of PFOS, indicating a loss in fluorine signal. This loss in fluorine is accounted for in two ways and has been reported in other electrochemical PFAS degradation studies.^[^ ^10^, ^17^, ^18^, ^19^ ^]^ The first is partial mineralization to fluoride and tetrafluoroborate ions, the latter of which was detected in ^19^F NMR in DMSO‐*d_6_*, while the former was detected when the NMR solvent was changed to D_2_O (Figures S11 and S12).^[^ ^20^ ^]^ While some fluoride is in solution, the evolution of volatile HF can occur when fluoride is exposed to protic sources.^[^ ^18^, ^21^ ^]^ As mentioned above, HF evolution is supported by the presence of BF_4_^−^ in the crude reaction mixture after electrolysis. A second process resulting in the loss of fluorine is the formation of volatile, likely short‐chain, organofluorine compounds.^[^ ^22^ ^]^ These compounds are difficult to detect directly when they are generated simultaneously as HF, but we were able to estimate their formation through a degradation approach discussed below.^[^ ^23^ ^]^

The conversion of PFOS to a distribution of PFCAs with chain lengths between 8 and 4 carbons was quantified using HPLC‐MS (Table 1). When 10 F mol^−1^ are passed, less than 1% residual PFOS reactant is detected. Approximately 60% of the initial PFOS concentration is found as PFCAs, of which PFOA is the most abundant chain length detected, comprising approximately 80 mol% of the degradation products. The 7‐ to 4‐carbon chain length PFCAs comprise the remaining 20 mol% of detected byproducts, the concentration of which decreases as chain length decreases. This trend is consistent with a one‐carbon‐at‐a‐time chain shortening mechanism, such as decarboxylation‐hydroxylation‐elimination‐hydrolysis (DHEH), which has been proposed for other oxidative PFAS destruction experiments.^[^ ^2^, ^24^, ^25^, ^26^, ^27^, ^28^, ^29^ ^]^ The high concentration of PFOA compared to the shorter chain length PFCAs suggests that oxidative desulfonation of PFOS to its carboxylate analog is the primary transformation, whereas the subsequent chain scission to shorter‐chain derivatives occurs as a secondary process. Approximately 50% of the molar amount of starting material is unaccounted for in HPLC‐MS quantitation, which is further supported by the quantitative ^13^C NMR discussed below.

**Table 1:** HPLC‐MS quantitation of PFCAs and PFSAs in reaction solution after 10 F mol^−1^ charge passed (40.0 µmol PFOS starting reactant). The error was determined from an average of measurements taken in duplicate. N/D = not detected. % of sum calculated based on the amount of quantified material.

PFCAs	PFOA	PFHpA	PFHxA	PFPeA	PFBA
**Number of C atoms**	8	7	6	5	4
**Average (mg)**	7.64 ± 0.02	1.19 ± 0.02	0.300 ± 0.002	0.093 ± 0.003	0.028 ± 0.002
**Average µmol**	18.5	3.27	0.955	0.352	0.131
**% of sum**	78.85	13.98	4.09	1.49	0.57

PFSAs	PFOS	PFHpS	PFHxS	PFPeS	PFBS
**Number of C atoms**	8	7	6	5	4
**Average (mg)**	0.09 ± 0.01	0.0166 ± 0.0005	0.007 ± 0.003	N/D	N/D
**Average µmol**	0.180	0.0369	0.0175	N/D	N/D
**% of sum**	0.79	0.16	0.07	–	–

**Figure 3:**
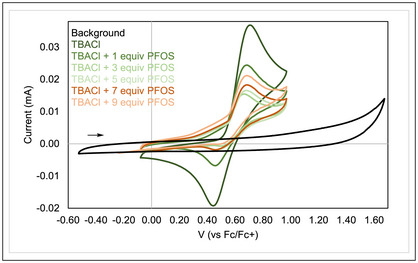
Cyclic voltammetry studies of chlorine and PFOS oxidation. Titrating a TBACl solution with increasing amounts of PFOS generates a catalytic increase in current from the regeneration of chloride ions. Conditions: 0.1 M TBABF_4_ in MeCN; 1 mM TBACl; N_2_ atmosphere; Pt WE, Pt CE, and Ag wire pseudo RE; 250 mV s^−1^ scan rate. The background trace consists of electrolyte solution only.

Cyclic voltammetry (CV) studies determined that the primary mechanism of PFOS conversion to PFCAs is oxidative. We explored the redox activity of PFOS in the presence of TBACl to assess the potential involvement of the electrolyte anion (Figure 3). The complete solubility of TBACl in acetonitrile enabled accurate dosing of chloride ions in solution for the CV study. We found that chloride oxidation to chlorine radical occurs at 0.5 V versus Fc/Fc^+^. The addition of one or three equivalents of PFOS to this solution initially decreased the current magnitude, likely because the introduction of PFOS at these concentrations leads to electrode passivation.^[^ ^30^, ^31^, ^32^ ^]^ Consecutive additions of PFOS to this solution generate a catalytic anodic wave, suggesting that Cl^—^ serves as a redox mediator in the oxidation of PFOS.^[^ ^33^, ^34^ ^]^ The same catalytic trend was observed with NaCl as a chloride source, and oxidation of PFOS without chloride present was very minor (Figure S3a,b). Adding increasing amounts of PFOS to water did not significantly change the current magnitude of the water oxidation peak (1.1 V versus Fc/Fc^+^) but instead generated a small oxidation peak at about 0.6 V versus Fc/Fc^+^ (Figure S3c). In these experiments, the increased solubility of PFOS in aqueous solutions may enable more efficient oxidation. The limited effect of PFOS on water oxidation indicates that its oxidation mediated by hydroxyl radicals is sluggish. Water oxidation outcompetes chloride and PFOS oxidation under aqueous conditions, indicating that this chloride‐mediated oxidation of PFOS is unique to organic solvent (Figure S3d).

**Figure 4:**
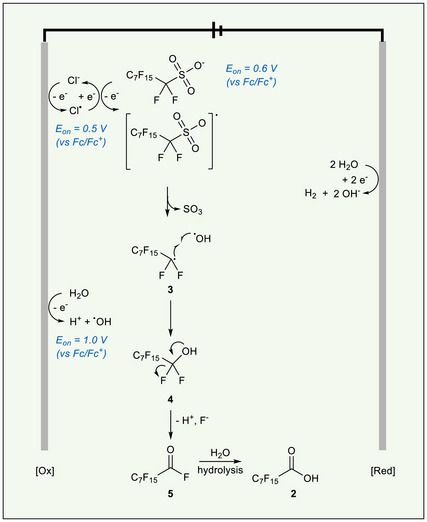
Proposed mechanism for conversion of PFOS to PFOA. Chloride mediates the oxidation of PFOS to a carbon‐centered radical, which couples with a hydroxyl radical to generate **4**. This intermediate undergoes elimination to generate acid fluoride, which is hydrolyzed to form PFOA (**2**). *E*_on_, onset oxidation potential values. [O*x*], electrochemical oxidation processes; [Red], electrochemical reduction processes.

Overall, these CV results suggest the primary mechanism for the electrochemical conversion of PFOS to PFOA is oxidation mediated by chloride. Trace water in MeCN may improve PFOS solubility and the overall reaction rate. This interpretation is bolstered by the result of an H‐cell experiment, in which PFOS‐containing solutions under the above standard conditions were electrolyzed on each side of an H‐cell separated by a Fumasep membrane. The anodic side of the cell showed BF_4_^−^ formation, while the cathodic side did not (Figure S2). Based on these findings, we propose that the transformation of the sulfonate group to a carboxylic acid begins with the chloride‐mediated, single‐electron oxidation of PFOS to generate carbon‐centered radical **3** at the anode.^[^ ^8^, ^35^, ^36^, ^37^ ^]^ This radical combines with a hydroxyl radical to generate **4**, which undergoes elimination to form acid fluoride **5**. The acid fluoride undergoes hydrolysis in the presence of water to generate PFOA (**2**). On the cathodic side, we propose that trace water undergoes proton reduction as the counter reaction for PFOS oxidation (Figures 4 and S3a).

As mentioned above, the observation of tetrafluoroborate and fluoride suggested the generation of HF during the reaction, which would reduce the overall fluoride recovery. Ion chromatography after DMSO/NaOH destruction of the post‐electrolysis reaction mixture also confirmed low fluoride recovery, with approximately 40% of the theoretical maximum fluoride detected. We would expect these strongly alkaline conditions to degrade the tetrafluoroborate anion to fluoride so the formation of this species likely does not affect the ultimate fluoride recovery (see Supporting Information p.9). We confirmed the reaction solution acidifies over time and confirmed the generation of volatile HF by suspending a pH indicator‐stained silica thin layer chromatography plate (TLC) in the reaction headspace (see SI p. 6–7). We also performed X‐ray photoelectron spectroscopy (XPS) of the TLC plate and the Pt electrodes after the reaction (Figures S5–S7). XPS showed a prominent peak at 686.9 eV, attributed to the F(1s) binding energy consistent with a Si─F bond formed via etching of silica by HF generated during the reaction.^[^ ^21^ ^]^ The F(1s) trace of the Pt electrodes after the standard washing procedure indicated an increased presence of an F‐containing species on the electrode surface for the control reaction with no current compared to the plate from the standard reaction conditions. However, the binding energy of this peak, 687–688 eV, is not consistent with a Pt─F bond and instead corresponds to that expected for C─F*_x_* bonds.^[^ ^38^ ^]^ Overall, pH analysis of the reaction medium and detection of Si─F bonds generated within the headspace of the reaction highlights the loss of F as HF.

The PFCAs produced as the major byproducts of the electrooxidation were mineralized under conditions previously reported by Dichtel and coworkers.^[^ ^2^ ^]^ Heating PFAS containing a carboxylic acid head group or at least one C─H bond to 120 °C in DMSO with NaOH degrades them to fluoride and benign non‐fluorinated carbon byproducts. This destruction reaction was monitored by ^19^F NMR spectroscopy to confirm the loss of all fluorine signals corresponding to PFCAs and the production of trifluoroacetate (TFA), a known byproduct of the mineralization reaction. To determine the carbon mass balance after the reaction, ^13^C NMR spectroscopy was used to identify and quantify known byproducts using an internal standard of potassium acetate (referenced to 181.2 ppm) in D_2_O (Figure S13). The major carbon products were identified as carbonate (168.1 ppm), oxalate (172.6 ppm), and formate (171.3 ppm). Integrating these signals against the signal corresponding to a known concentration of potassium acetate enabled the quantification of the carbon byproducts and calculation of the carbon mass balance for PFOS mineralization. Carbon recovery of approximately 60% compared to the theoretical maximum, which assumes that all the carbon in PFOS is converted to quantifiable carbon byproducts detectable through NMR spectroscopy, was obtained after mineralizing the PFCAs generated from the electrochemical desulfonation of PFOS. This result, paired with the quantitation of PFCAs from HPLC‐MS, indicates incomplete carbon and fluorine balances during electrochemistry, which is attributed to the formation of volatile compounds.

Characterization of the electrode surface following electrochemistry revealed the generation of a dark‐colored precipitate. The solid is soluble in water and dissolves during the electrode washing process, indicating that it is not a long chain perfluoroalkyl species that might arise by coupling two perfluoroalkyl chain radicals following oxidative desulfonation. Therefore, the most likely hypothesis to explain the large loss of carbon is the generation of volatile, short chain organofluorine species.^[^ ^39^ ^]^ As mentioned above, the determination of volatile species through headspace GC‐MS is complicated by the simultaneous production of volatile HF. To prevent HF generation and search for these potential fluorinated byproducts, the reaction was performed with tetramethylammonium hydroxide pentahydrate (TMAOH) as an electrolyte. TMAOH produces a similar distribution of PFCAs as NaCl, albeit with inferior efficiency, with 80% conversion of PFOS after 10 F mol^−1^ is passed. Performing the same headspace pH analysis using a suspended, pH indicator‐stained Si TLC plate showed no evidence for the formation of volatile HF (see Supporting Information p. 6–7). Under these conditions, several low molecular weight species, ranging between *m*/*z* values of 50 to 250, were detected by headspace GC‐MS (Figure S14). These volatile species range between one and four carbons and can contain between three and nine fluorine atoms. The significant generation of these volatile organofluorine compounds is consistent with both the modest carbon balance (42%) and fluorine balance (61%) from the electrolysis of PFOS.

The standard electrochemical conditions were applied to perfluorohexane sulfonate (PFHxS) and perfluorobutane sulfonate (PFBS), the 6‐ and 4‐carbon PFSAs, respectively, to evaluate their conversion into perfluoroalkyl carboxylic acids (Figures S15–S16). The electrochemical oxidation of PFHxS produced PFCAs containing between two and five carbons as major products, while PFBS produced PFCAs containing between two and four carbons, as detected by ^19^F NMR spectroscopy. These compounds appear to follow the same reactivity as PFOS via a sulfonate to carboxylate mechanism, where PFCA products with the same number of carbons (eight in the case of PFOS) and lower are produced from the reaction. In contrast to the reaction with PFOS however, the conversion to PFCAs is observed to decrease with shorter chain length. Out of the 8‐, 6‐, and 4‐carbon PFSAs, PFBS was the most difficult to desulfonate, as indicated by the large amount (50%) of starting material remaining after the reaction. This lack of conversion is partially attributed to the continuous fouling of the electrode during the reaction, as the measured current in solution was observed to increase over time. To mitigate this issue, alternating polarity (AP) with a frequency of 0.5 Hz was applied during the reaction to attempt to prevent the accumulation of short‐chain PFAS on the electrodes.^[^ ^40^ ^]^ The application of AP improved the observed current during the reaction but did not ultimately increase the conversion of PFBS to PFCAs.

## Conclusion

We have identified a simple, non‐aqueous electrochemical method for the conversion of PFSAs to PFCAs that enables their downstream mineralization to primarily fluoride, trifluoroacetate, carbonate, oxalate, and formate. A robust mechanistic study using PFOS as a model substrate suggests that the reaction proceeds through an initial oxidative desulfonation, mediated by a chlorine radical, to a perfluorinated alkyl radical and subsequent coupling with a hydroxyl radical. This process is followed by elimination and hydrolysis to generate PFOA. These conditions also degrade 4‐ and 6‐carbon chain PFSAs, though further study is needed to promote complete conversion to their corresponding PFCAs. Ultimately, this work investigated the electrochemical reactivity of PFSAs under oxidative conditions to establish the mechanism of head group conversion in organic solvent. This method can achieve near‐complete PFOS desulfonation; however, the generation of volatile organofluorine products would need to be suppressed before these conditions are adapted as a competitive PFAS destruction technology. Nevertheless, the demonstration that chloride from simple NaCl salt is an effective redox mediator for perfluoroalkylsulfonate degradation under accessible electrochemical conditions is an exciting breakthrough for the green chemistry community and will inspire further investigation into the fundamental mechanisms underlying PFAS degradation in organic solvents.

## Supporting Information

The authors have cited additional references within the Supporting Information.^[^ ^41–45^ ^]^

## Conflict of Interests

The authors declare no conflict of interest.

## Supporting information



Supporting Information

## Data Availability

The data that support the findings of this study are available in the Supporting Information of this article.
