# Supplementary material for: Electrochemical Degradation of Perfluoroalkyl Sulfonates via Sulfonate to Carboxylate Conversion
Source: Angew Chem Int Ed Engl. 2026 Jan 9;65(7):e25896. doi: 10.1002/anie.202525896 (PMC12887609; doi:10.1002/anie.202525896)
Supplement: Supplementary file 1 — Supporting Information [file ANIE-65-e25896-s001.pdf]

Electrochemical Degradation of Perfluoroalkyl Sulfonates via Sulfonate to Carboxylate Conversion

Stella A. Fors†, Richard J. Monsky†, Emily R. Mahoney,
Christian A. Malapit*, William R. Dichtel*

Department of Chemistry, Northwestern University, 2145 N Sheridan Rd, Evanston, Illinois 60208, USA

†These authors contributed equally.

*Corresponding authors' email addresses:
christian.malapit@northwestern.edu; wdichtel@northwestern.edu

Contents

General information 2
Procedures 2
Optimization 3
Mechanistic Studies 5
NMR Spectra 15
References 24

General information

All reagents and solvents were used as purchased from suppliers, unless stated otherwise. Acetone and acetonitrile were purchased from Fisher Chemical, and anhydrous acetonitrile was purchased from Thermo Fisher. All deuterated solvents were purchased from Cambridge Isotope Laboratories Inc. PFAS derivatives were purchased from Synquest Labs.

Cyclic voltammetry was performed under a N_2 atmosphere (unless otherwise stated) using a Biologic VSP multichannel potentiostat/galvanostat. A three-electrode set-up was employed, including a 3.0 mm diameter glassy carbon disc working electrode (BASi, MF-2012), or a 3.0 mm platinum disc working electrode (BASi, MF-2113), platinum wire counter electrode, and a silver wire pseudo reference electrode. The working electrode was polished in between each data collection on a microcloth pad. Measurements were performed using a 0.1 M electrolyte solution, with 1-5 mM analyte and a scan rate of 10-500 mV/s. Voltammograms were reported as V vs. Fc/Fc+.

NMR spectroscopy was performed using a Bruker Avance III HD 500 spectrometer operating at 500 MHz and 471 MHz for ^{1}H and ^{19}F NMR experiments, respectively, and analyzed using MestreNova compared to synthesized standards or literature values. X-ray Photoelectron Spectroscopy (XPS) was performed using a Thermo Scientific ESCALAB 250 Xi. Thin layer chromatography (TLC) staining was performed using EMD TLC plates pre-coated with 250 µm thickness silica gel 60 F254 plates and 0.1% bromocresol green solution in ethanol. HPLC-MS was performed on an Agilent 6475 LC/TQ triple quadrupole instrument coupled to an Agilent Infinity II 1290 UHPLC instrument.

The IKA Electrasyn 2.0 was used for all electrochemical reactions (undivided, glass cell) including optimization by varying the solvent, supporting electrolyte, electrode material, and amount of charge passed. Further details are provided below regarding Electrasyn settings. Pt plates and wire were purchased from Goodfellow Cambridge Ltd. Pt plates were cut into 1 cm^2 pieces and hooked through 1-2 inches of Pt wire then secured to the Electrasyn electrode holders with aluminum foil. Pt electrodes were adjusted to be of similar length and parallel to each other with a gap of approximately 1 cm in between. Reticulated vitreous carbon (RVC) electrodes were cut into 5 cm x 1 cm x 0.3 cm strips from a 6 in x 6 in sheet (100 ppi, 0.5 in thickness) purchased from Duocell and secured to the Electrasyn cap using the electrode holders. A rubber septum was secured on the inlet of the cap. All vials, caps, electrode holders, and stir bars were purchased from IKA. No reference electrodes were used.

Procedures

General procedure, work up, and analysis:

Potassium perfluorooctane sulfonate (0.04 mmol) and electrolyte (7.7 equiv) were weighed into a 5 mL Electrasyn vial containing a Teflon stir bar and with Teflon wrapped threads. Solvent (5 mL) was added to the vial via a syringe. The vial was stirred until the solid was dissolved. The Electrasyn cap was fitted with electrodes and secured onto the vial. The solution was electrolyzed.

After the reaction was complete, the electrodes were rinsed with solvent and sonicated twice: first in water, then fresh reaction solvent, each for 10 minutes. The washes were combined and evaporated before being dissolved in deuterated solvent for analysis.

Optimized reaction procedure:

Potassium perfluorooctane sulfonate (PFOS, 0.04 mmol) and NaCl (7.7 equiv) were weighed into a 5 mL Electrasyn vial containing a Teflon stir bar and with Teflon wrapped threads. MeCN (5 mL) was added to

the vial via a syringe. The vial was stirred until the solid was dissolved. The Electrasyn cap was fitted with Pt plate electrodes (secured into the electrode holders with Al foil and Pt wire) and screwed onto the vial. The solution was electrolyzed at 40 mA for 10 F/mol using the IKA Electrasyn 2.0.

IKA Electrasyn Set Up:

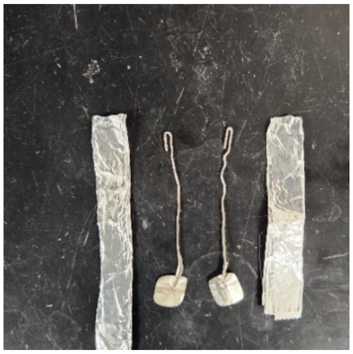

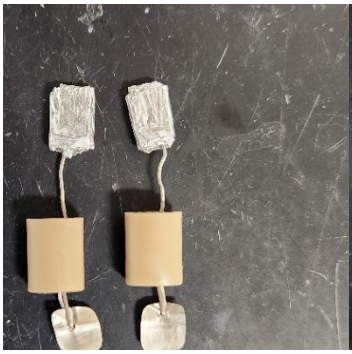

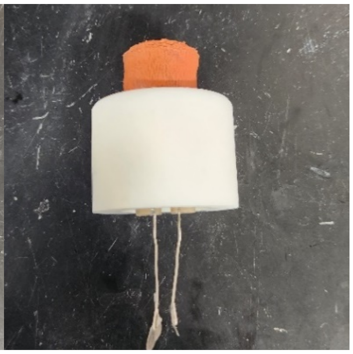

(a) (b) (c)

a) Pt electrodes and aluminum foil
b) Insertion of electrodes into adapters
c) Completed cap set up

Short chain PFAS procedure:

Potassium perfluorohexane sulfonate (PFHxS) or potassium perfluorobutane sulfonate (PFBS) (0.04 mmol) and NaCl (7.7 equiv) were weighed into a 5 mL Electrasyn vial containing a Teflon stir bar and with Teflon wrapped threads. MeCN (5 mL) was added to the vial via a syringe. The vial was stirred until the solid was dissolved. The Electrasyn cap was fitted with Pt plate electrodes (secured into the electrode holders with Al foil and Pt wire) and screwed onto the vial. The solution was electrolyzed at 40 mA for 50 F/mol with a 0.5 Hz rapid alternating polarity frequency using the IKA Electrasyn 2.0.

Optimization

Solvent

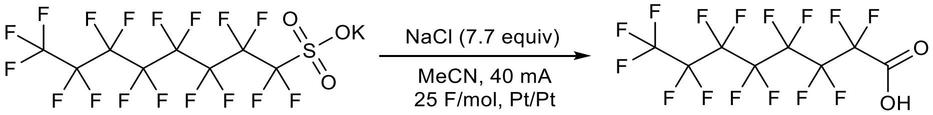

Change from Above	PFOS Conversion (%)
none	> 95
Acetone, RVC	90
Sulfolane	< 5
DMA	< 5

F/mol

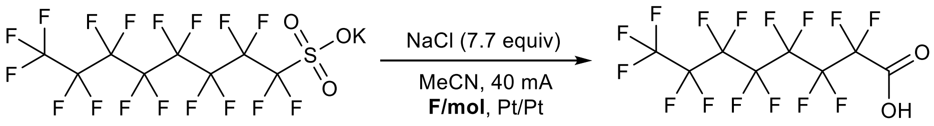

Charge Passed (F/mol)	PFOS Remaining (%)
0	100
0.5	45
1	38
2.5	17.2
5	2.7
10	1.5
25	1.5

Electrolyte

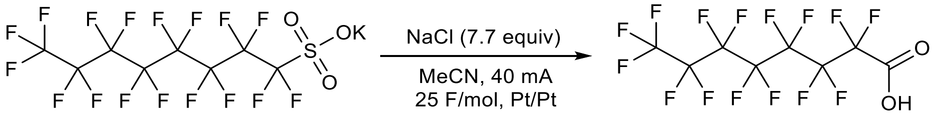

Change from Above	PFOS Conversion (%)
none	> 95, very high resistance
$TBAPF_6$	< 5
NaCl, Acetone, RVC, 50 F	Cannot determine
NaBr, Acetone, RVC, 50 F	Cannot determine
$NaClO_4$, Acetone, RVC, 50 F	Cannot determine
TMAOH • 5 H_2O (10 equiv)	80
TMAOH • 5 H_2O (5 equiv), RVC	0
TBACl	0

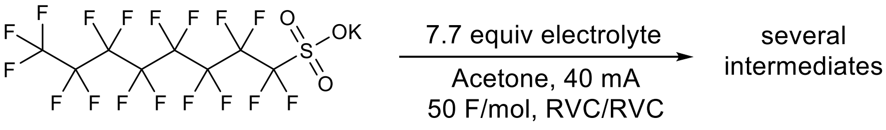

0.04 mmol

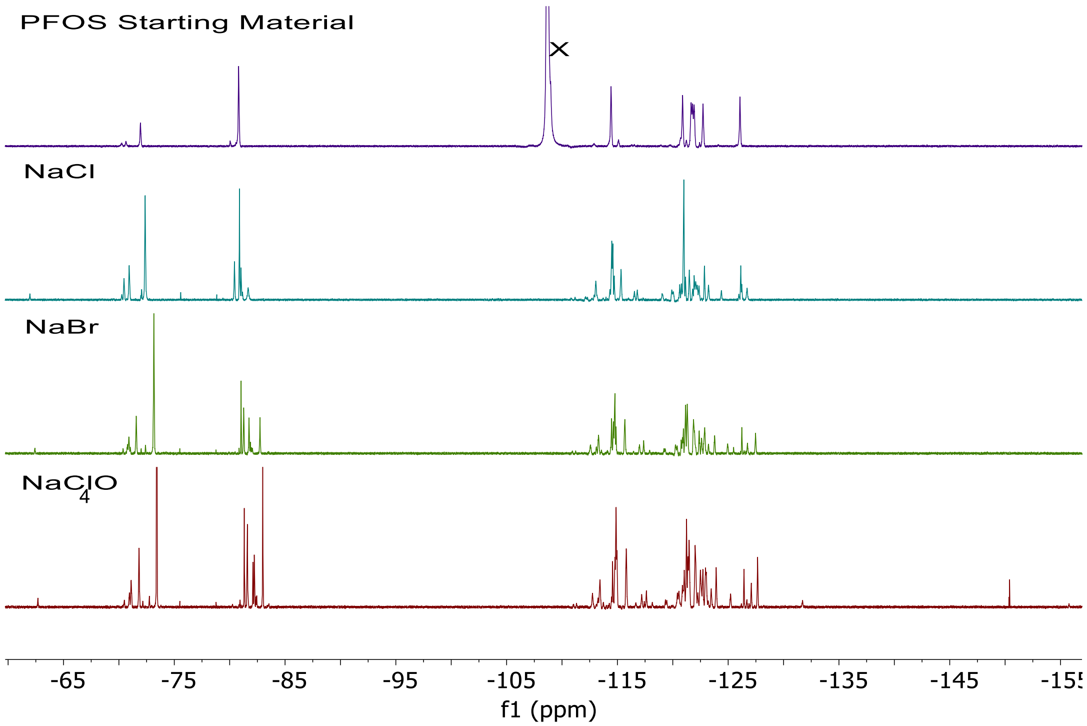

Figure S1: ^{19}F NMR spectra from preliminary reaction conditions employing acetone, RVC electrodes, and various electrolytes. The spectra highlight the conversion of PFOS to multiple indistinguishable intermediates. Conditions with NaCl qualitatively indicate the most fluoride produced (~ –120 ppm), suggesting the most degradation. Later optimization to standard conditions with acetonitrile and Pt led to a more controlled mechanism, primarily resulting in the conversion of PFOS to PFOA.

Electrode Material

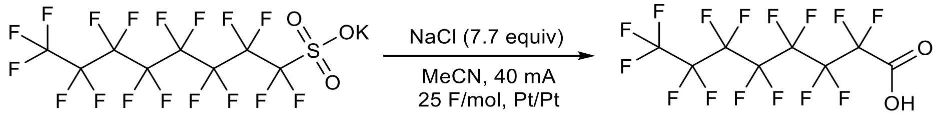

Change from Above	PFOS Conversion (%)
none	> 95
RVC	90
Ni Foam	0
Graphite	45
Stainless Steel	0
Pt/Zn (sacrificial anode)	< 5
Pt/Mg (sacrificial anode)	0

Mechanistic Studies

Gas phase acid detection via stained TLC plate:

The general procedure for adding reagents and solvent was followed above, but 4 mL of MeCN was used. An approximately 1 x 1 cm^2 silica TLC plate was stained using 0.1% Bromocresol green solution in ethanol. After the Pt electrodes were fastened onto the Electrasyn cap, the plate was glued onto the bottom of the electrode holder using liquid rubber. The glue was allowed to dry for 5 minutes before fastening the cap onto the Electrasyn vial and running the reaction. The color of the TLC plate was recorded before starting the electrolysis and after the reaction started. The TLC plate was always blue after initial staining (see **a** below).

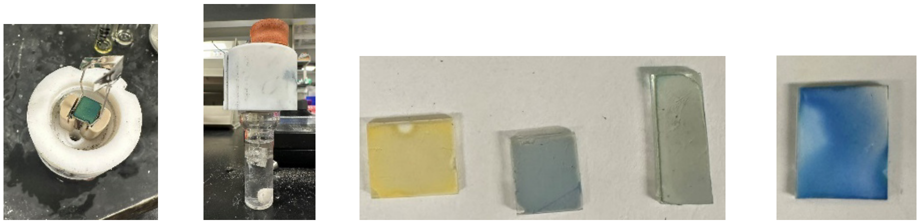

a b c d

(a) Appearance after staining and gluing to inside of cap (before reaction)
(b) Overall set up (plate is within cap so cannot be seen)
(c) Plate appearance after reaction conditions, left to right: optimal conditions, no electricity, CsF (no PFOS or NaCl)
(d) Plate appearance after reaction using TMAOH instead of NaCl.

Time point acid-base indicator and F^-/HF detection tests:

The procedures were followed as described above. At 0 F, 8 F, 16 F, and 25 F/mol (0 for CsF) a 100 uL aliquot was removed from the reaction in place in a vial. Deionized water was added to approximately double the volume of the aliquot. A drop of bromocresol green solution (0.1% in ethanol) was added and the vial was gently shaken. The color of the solution was recorded.

The optimal procedure was followed as described above. At 0 F, 8 F, 16 F, and 25 F/mol, a 100 uL aliquot was removed from the reaction in place in a vial. Deionized water was added to approximately double the volume of the aliquot. Two drops of the sample were placed on the test strip. The strip was assessed within 2 minutes of adding the sample. A white ring indicates the presence of F^-/HF in the solution.

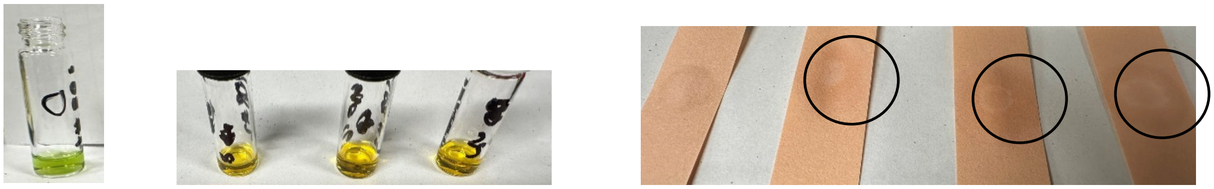

a b c

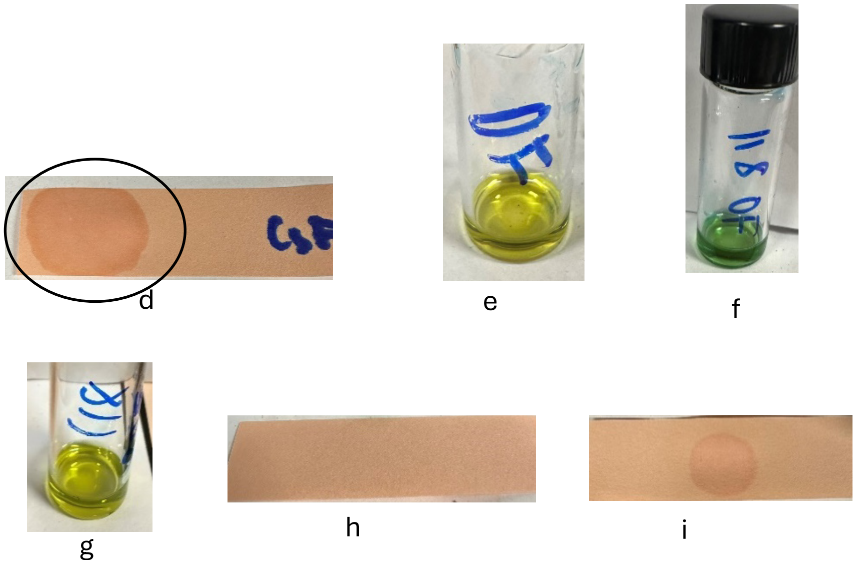

(a) Indicator test for reaction mixture at 0 F/mol (neutral)
(b) Indicator test for reaction mixtures at 8 F, 16 F, and 25 F/mol (acidic)
(c) Test strip appearance for reaction mixtures at 0 F, 8 F, 16 F, and 25 F/mol (left to right) with the fluoride indication rings circled
(d) Control test strip appearance (CsF solution) with fluoride indication ring circled
(e) Indicator test for reaction mixture with just CsF at 0 F/mol (initial, acidic)
(f) Indicator test for reaction with no PFOS (just NaCl) at 0 F/mol (neutral)
(g) Indicator test for reaction with no PFOS (just NaCl) at 25 F/mol (slightly acidic)
(h) Test strip appearance for reaction with no PFOS (just NaCl) at 0 F/mol
(i) Test strip appearance for reaction with no PFOS (just NaCl) at 25 F/mol

H-Cell Experiment:

Potassium perfluorooctane sulfonate (0.04 mmol) and NaCl (7.7 equiv) were weighed into both sides of an H-cell (5 mL) containing an Fumasep membrane. 5 mL of MeCN were added to each side using a syringe and the solutions were stirred for about 5 minutes until the solid was dissolved. Each side was fitted with a Pt plate electrode secured to a glass holder with Cu wire. The H-cell was sealed and connected to an ElectraSyn 2.0 using alligator clips to set the parameters for the experiment, applying 40 mA for 20 F/mol.

After reaction completion, ^{19}F NMR of the crude reaction solutions was used to assess PFOS conversion and generation of PFOA on each side of the H-cell. The bifluoride peak (about –150 ppm) present in the spectrum from the anodic side of the H-cell suggests effective partial conversion of PFOS. Meanwhile, the spectrum from the cathodic side of the cell does not indicate the presence of bifluoride, suggesting minimal degradation. This result confirms the mechanism of the reaction is primarily oxidative.

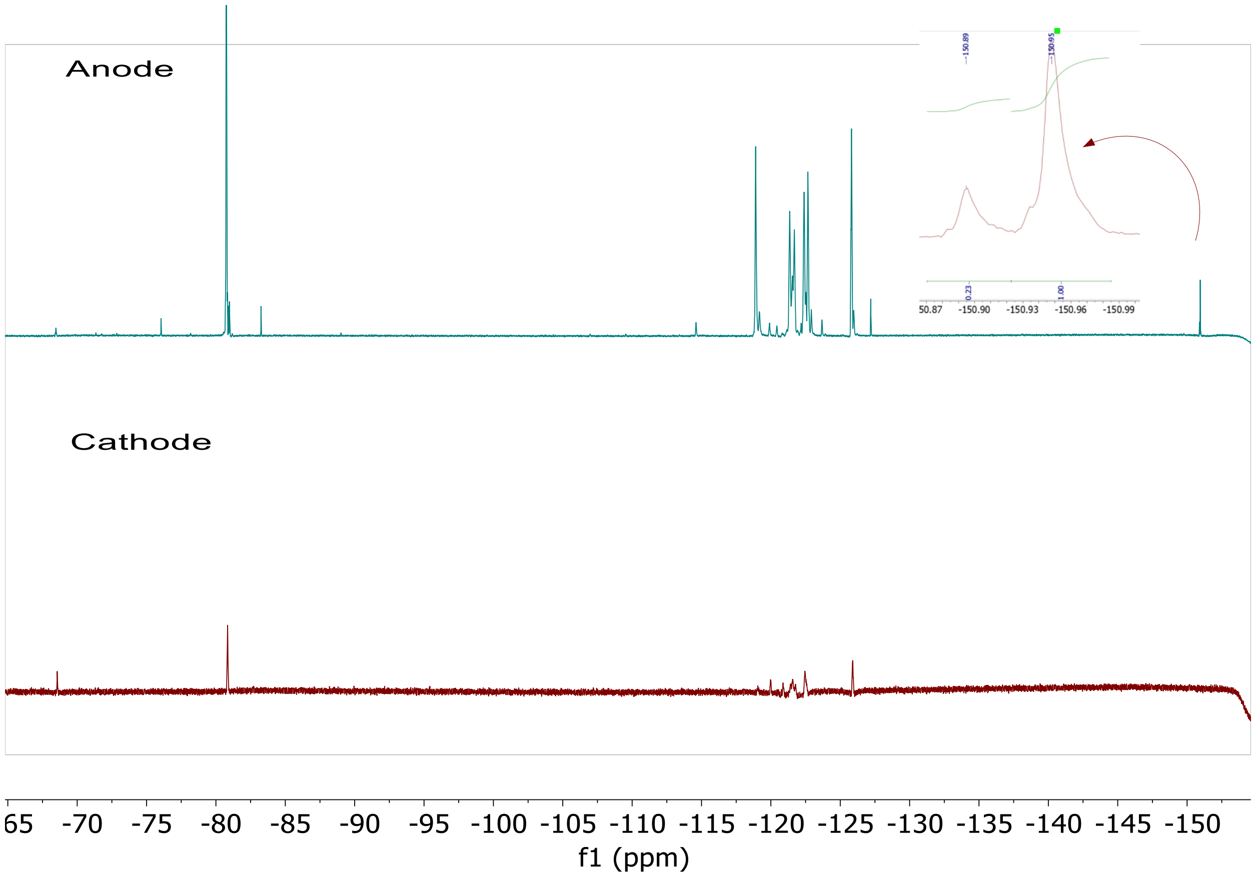

Figure S2. ^{19}F NMR spectra from anodic and cathodic side of an H-cell. Ideal reaction conditions were performed on both sides of an H-cell to assess whether the primary mechanism for conversion of PFOS to PFOA is oxidative or reductive. A zoomed profile showing the peaks at –150 ppm and their ~1:4 integration (0.23:1) identify it as the tetrafluoroborate anion, which is likely formed by a reaction between HF and the borosilicate glass of the H-cell and/or NMR tube.[41] This species would not contribute to the modest fluoride recovery post base degradation, as BF_4^- would likely degrade to fluoride in the presence of water and under strongly basic and heated conditions.[42]

Electrolysis in Polyethylene Vial:

The optimized procedure for PFOS degradation was followed using a 20 mL polyethylene vial. Approximately 10 mL of MeCN was used to submerge the electrodes. ^{19}F NMR of the crude reaction mixture after evaporation and subsequent dissolution in DMSO-d_6 shows trace BF_4^-, from HF etching the borosilicate NMR tube glass. Two ^{19}F NMR spectra of the reaction post electrolysis are included at the end of the NMR Spectra section.

Cyclic Voltammetry:

CVs in Figures S3a-3c and Figure S4 were performed with 0.1M $TBABF_4$ in MeCN under N_2 atmosphere and with a Pt WE, Pt CE, and Ag wire pseudo RE. 250 mV/s scan rate and three scans. The WE was polished between each collection. The third scan is shown for each curve. CVs in Figures S3d was

performed in water (more detail below), but with the same electrode set up and scanning conditions as the other CVs.

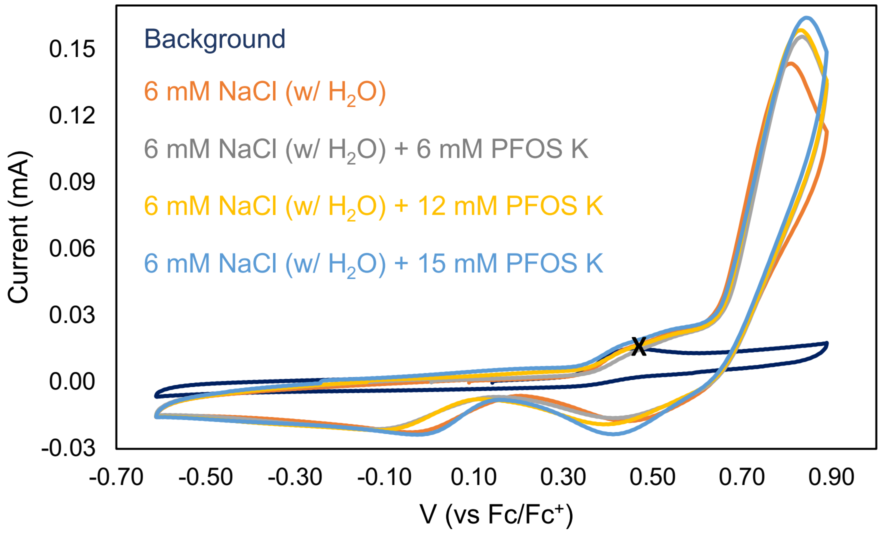

Figure S3a: CV indicating catalytic effect of NaCl on PFOS oxidation, with added water to solubilize NaCl (240 uL) in the $TBABF_4$/MeCN solution.

The oxidation wave belongs to the oxidation of chloride to chlorine radical. Successive additions of PFOS generate a catalytic current, indicating the regeneration of chloride. The reductive wave beginning at about 0.1 V is likely the reduction of water.[43,44]

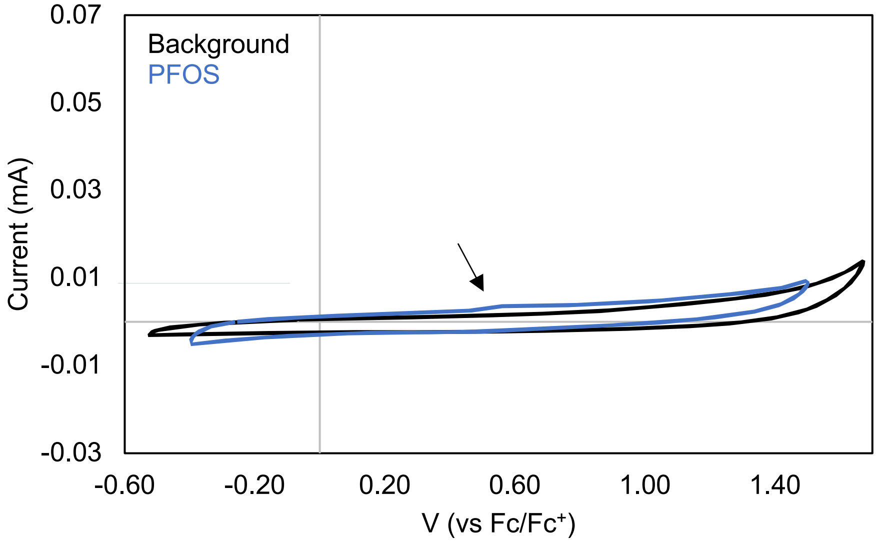

Figure S3b: CV indicating minimal oxidation of PFOS (0.6 V vs Fc/Fc^+) without mediator present. Performed in $TBABF_4$/MeCN solution.

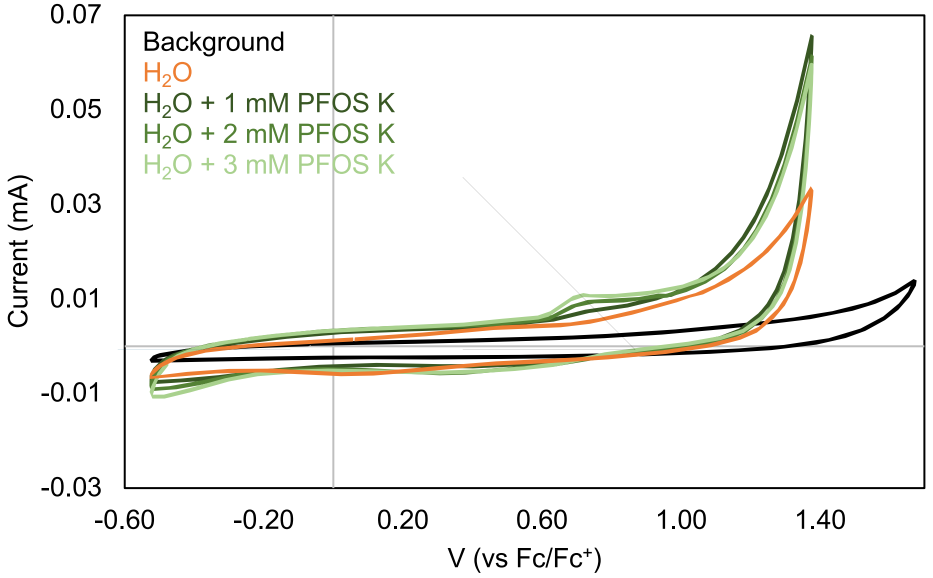

Figure S3c: CV indicating small catalytic effect of water on PFOS oxidation. A small oxidation peak is generated at about 0.6 V (vs Fc/Fc^{+}), suggesting the added water solubilizes PFAS in the $TBABF_4$/MeCN solution. Successive addition of PFOS does not lead to further increase in current, suggesting the hydroxyl radical mediated oxidation of PFOS is a sluggish process. Because this process occurs at a greater oxidation potential than chloride oxidation, the chlorine radical mediated oxidation is likely the primary mechanism.

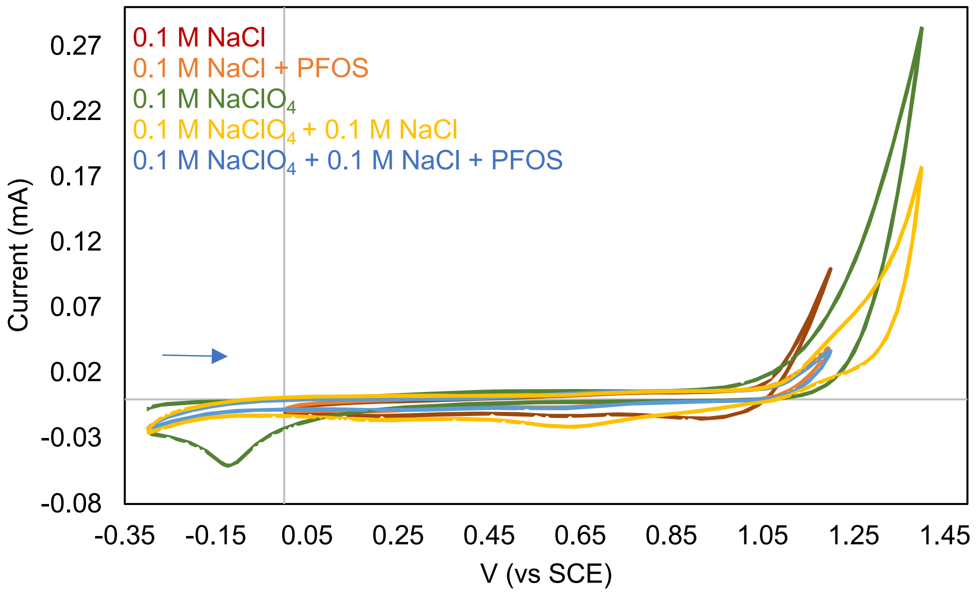

Figure S3d: CV highlighting system performance in water. CVs were performed in aqueous solutions and are referenced to SCE. Oxidation of water (about 1.1 V) is observed in all cases instead of Cl^- or PFOS oxidation, as evidenced by all peaks overlapping with the green trace (water and $NaClO_4$, a non-redox active electrolyte).

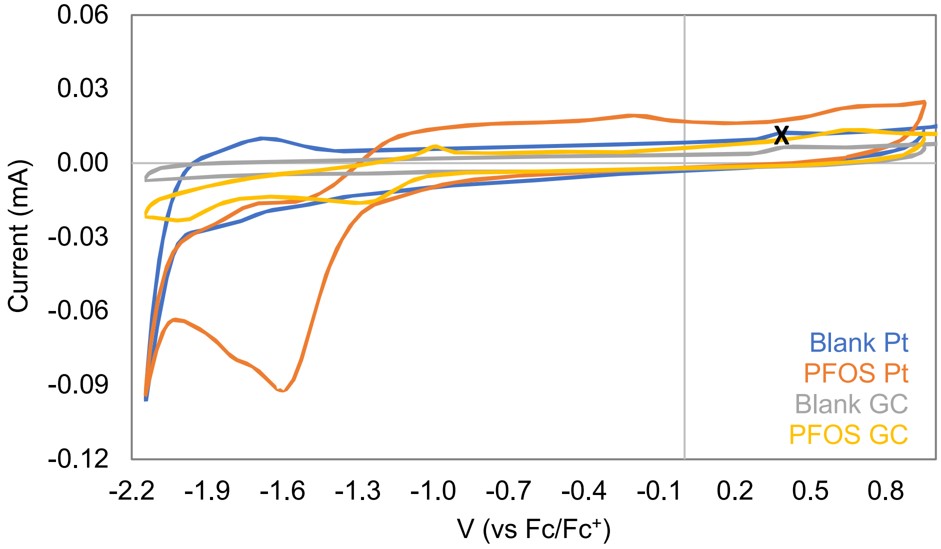

Figure S4: CV showing reduction wave of PFOS and effect of Pt WE vs GC WE. Performed in $TBABF_4$/MeCN.

X-Ray Photoelectron Spectroscopy:

The procedure described above for the stained TLC plate experiments was followed except for in one experiment the plate was left unstained. The standard reaction conditions were followed using NaCl.

The plate was dried in air at room temperature at least overnight before XPS measurements. Electrodes were subjected to the regular washing procedure, 10 min sonication in water and 10 min sonication in MeCN, before allowing them to dry in air at room temperature for at least 3 days before XPS measurements.

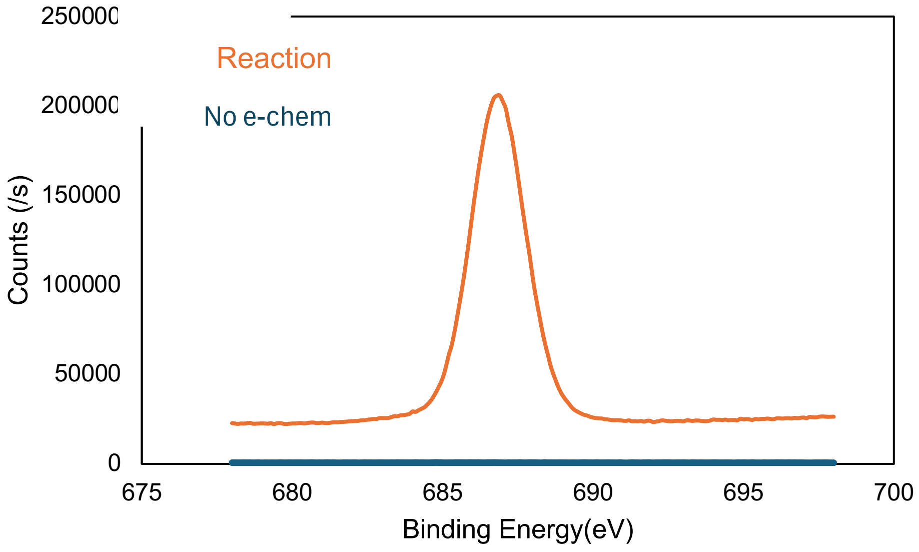

Figure S5: XPS spectrum of F(1s) on silica plate (stained)

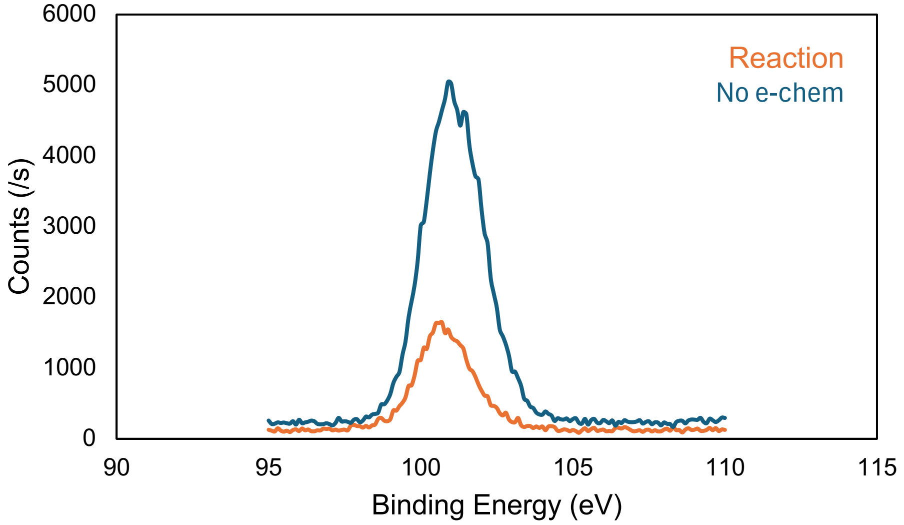

Figure S6: XPS spectrum of Si(2p) on silica plate (unstained)

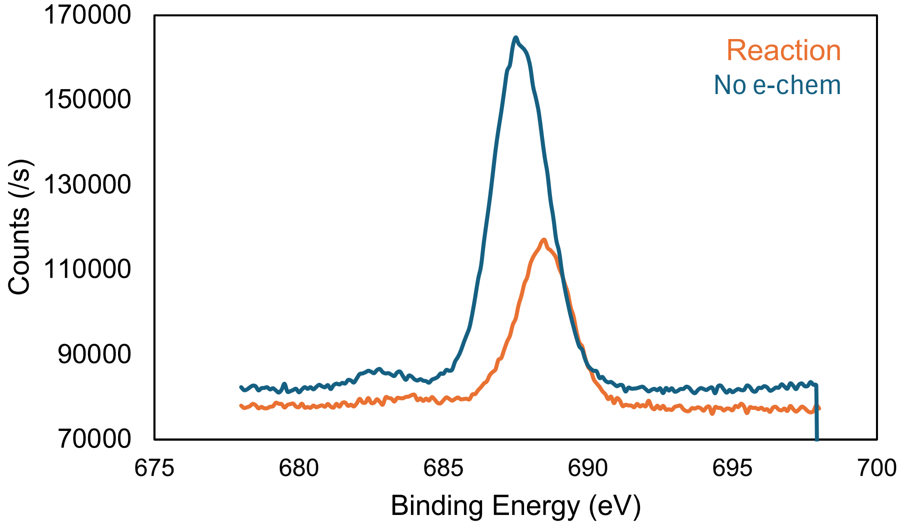

Figure S7: XPS spectrum of F(1s) on Pt electrode

Tracking current over time with and without NaCl:

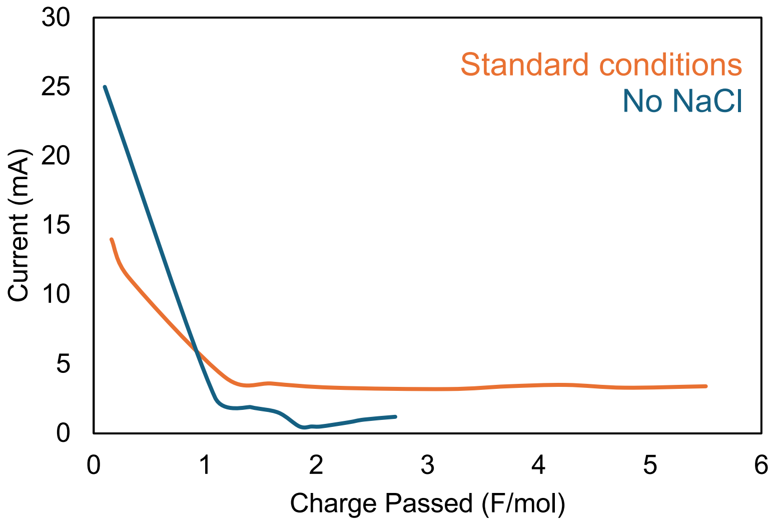

Figure S8: Current measured during reactions with and without NaCl at a set potential. The reactions were performed with PFOS at the standard conditions (with and without NaCl) set at 30 V rather than –40 mA, and current was recorded approximately every 10 minutes. The reaction without NaCl did not continue after about 3 F/mol passed because of high resistivity in the cell.

Reaction with excess base:

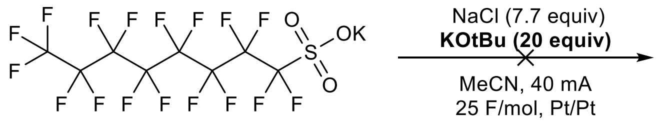

Potassium perfluorooctane sulfonate (PFOS, 0.04 mmol), NaCl (7.7 equiv), and potassium tert-butoxide (20 equiv) were weighed into a 5 mL Electrasyn vial containing a Teflon stir bar and with Teflon wrapped threads. MeCN (5 mL) was added to the vial via a syringe. The vial was stirred for 5 minutes until most of the solid dissolved. The Electrasyn cap was fitted with Pt plate electrodes (secured into the electrode holders with Al foil and Pt wire) and screwed onto the vial. The solution was electrolyzed at 40 mA for 25 F/mol using the IKA Electrasyn 2.0.

No reaction was observed and starting material was conserved.

Reactions with radical traps:

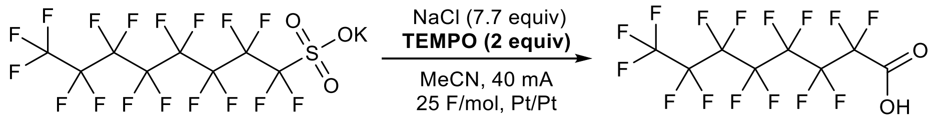

conversion w/o TEMPO **95%**

conversion w/ TEMPO **50%**

Potassium perfluorooctane sulfonate (PFOS, 0.04 mmol), NaCl (7.7 equiv), and TEMPO (2 equiv) were weighed into a 5 mL Electrasyn vial containing a Teflon stir bar and with Teflon wrapped threads. MeCN (5 mL) was added to the vial via a syringe. The vial was stirred for 5 minutes until the solid dissolved.

The Electrasyn cap was fitted with Pt plate electrodes (secured into the electrode holders with Al foil and Pt wire) and screwed onto the vial. The solution was electrolyzed at 40 mA for 25 F/mol using the IKA Electrasyn 2.0.

While conversion was reduced with TEMPO present, no TEMPO adduct was detected via NMR or GCMS, so we are unable to confirm the presence of a radical intermediate.

A similar procedure was followed using 5 equivalents of 1,1-diphenylethylene instead of TEMPO. PFOS conversion was also limited to 50%, suggesting 1,1-diphenylethylene inhibited radical reactivity, but no new products were detected via NMR.

Reaction with allyl alcohol (hydroxyl radical scavenger)[45]:

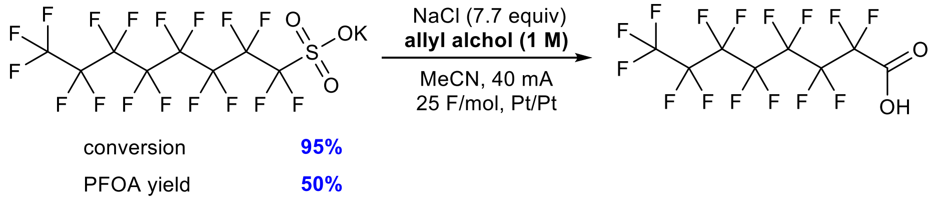

Potassium perfluorooctane sulfonate (PFOS, 0.04 mmol) and NaCl (7.7 equiv) were weighed into a 5 mL Electrasyn vial containing a Teflon stir bar and with Teflon wrapped threads. MeCN (5 mL) was added to the vial via a syringe. Allyl alcohol (1 M, 272 µL) was added with a micropipette. The vial was stirred for 5 minutes until most of the solid dissolved. The Electrasyn cap was fitted with Pt plate electrodes (secured into the electrode holders with Al foil and Pt wire) and screwed onto the vial. The solution was electrolyzed at 40 mA for 25 F/mol using the IKA Electrasyn 2.0.

While 95% of the PFOS starting material was converted, the reaction yielded 50% PFOA. This reduction in yield suggests the presence of hydroxyl radicals in the reaction solution, as allyl alcohol is a hydroxyl radical scavenger. We invoke hydroxyl radicals as the oxygen source for PFOA formation.

NMR Spectra

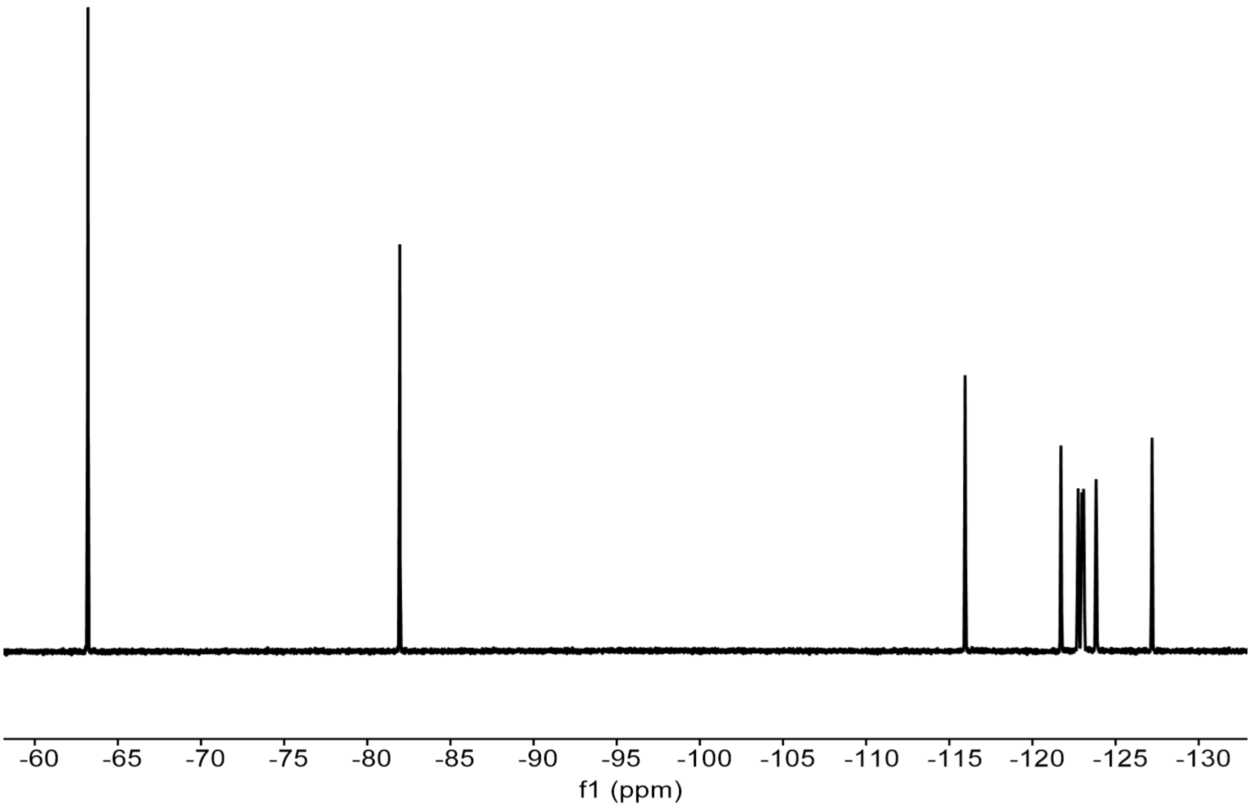

Figure S9. ^{19}F NMR spectrum (DMSO-d_6) of reaction mixture after electrolysis of PFOS in sulfolane with NaCl and Pt electrodes. Chemical shifts are referenced to α,α,α-trifluorotoluene at -63.2 ppm.

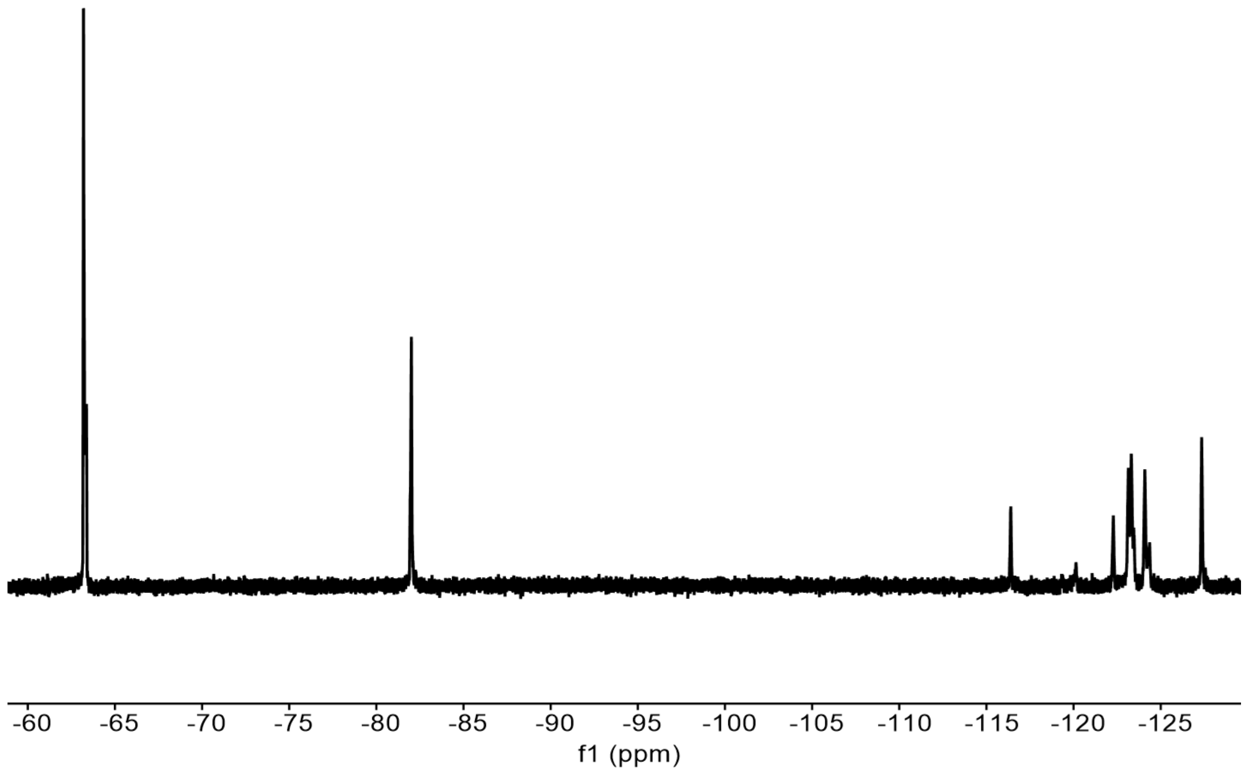

Figure S10. ^{19}F NMR spectrum (DMSO-d_6) of reaction mixture after electrolysis of PFOS in dimethylacetamide (DMAc) with NaCl and Pt electrodes. Chemical shifts are referenced to α,α,α-trifluorotoluene at -63.2 ppm.

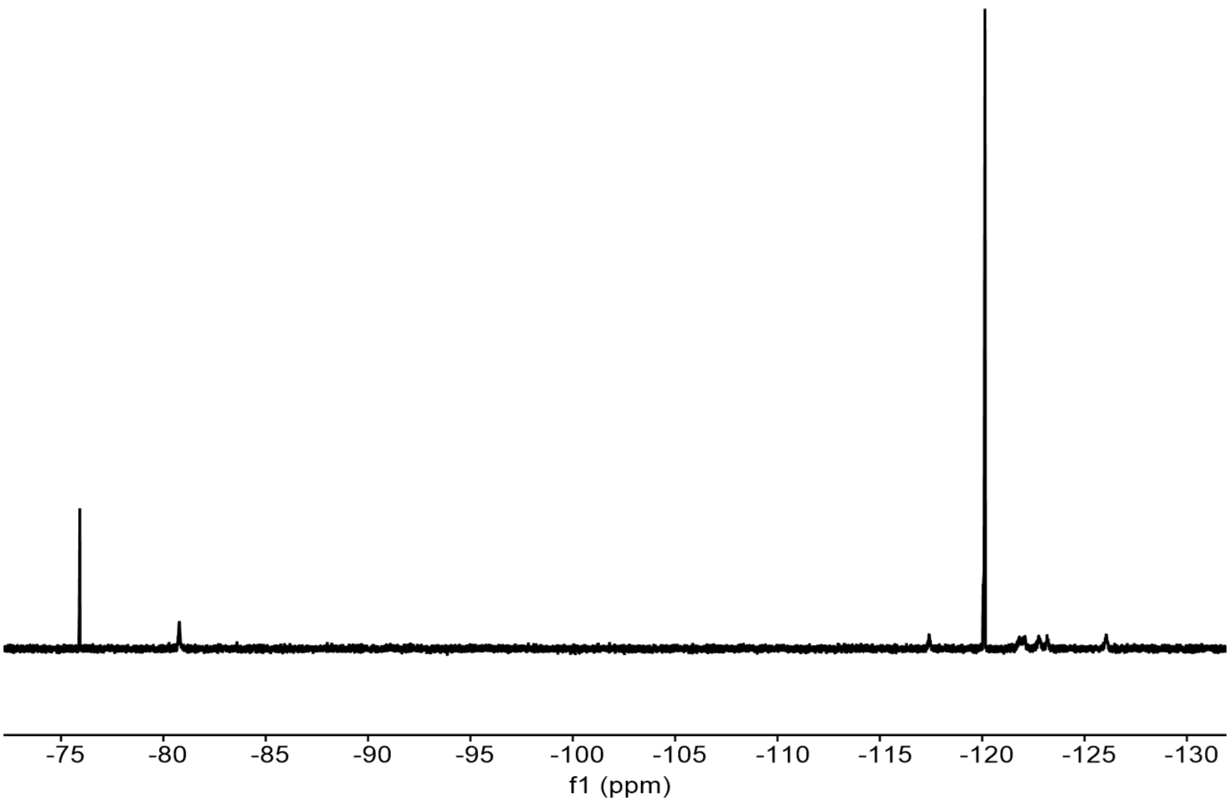

Figure S11. ^{19}F NMR spectrum (D_2O) of reaction mixture after electrolysis of PFOS in acetonitrile with NaCl. The prominent peak at -120.1 ppm corresponds to sodium fluoride (NaF).

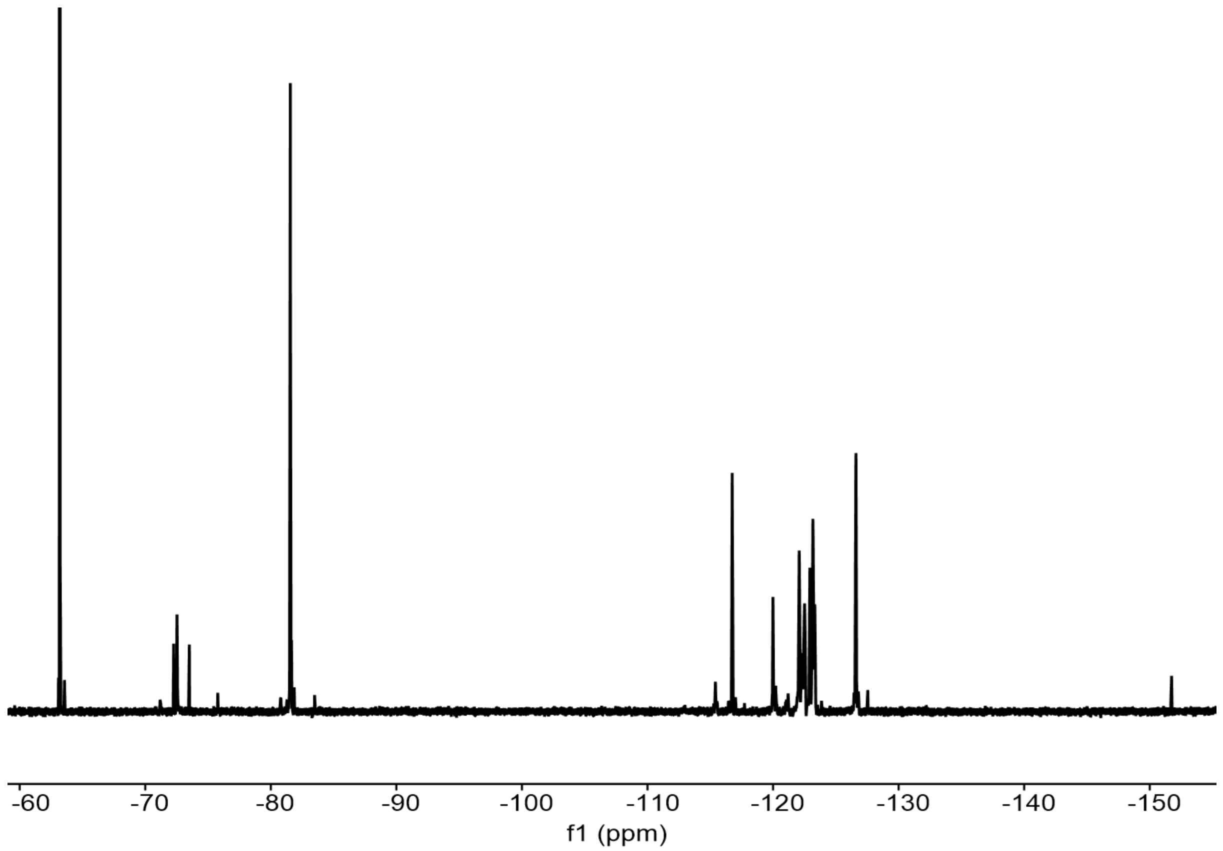

Figure S12. ^{19}F NMR spectrum (DMSO-d_6) of reaction mixture after electrolysis of PFOS in acetonitrile with NaCl and RVC electrodes. Chemical shifts are referenced to α,α,α-trifluorotoluene at -63.2 ppm.

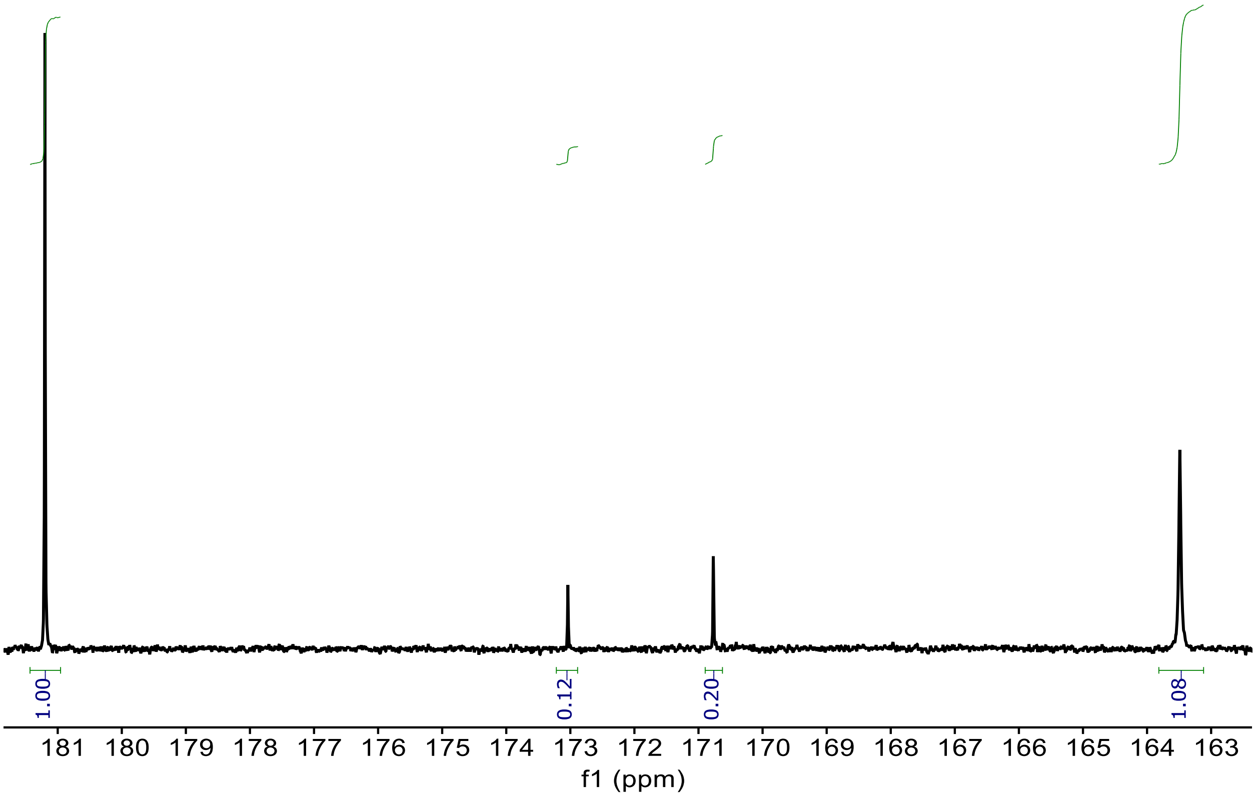

Figure S13. ^{13}C NMR spectrum (D_2O) of precipitate formed after DMSO/NaOH degradation of reaction mixture following electrolysis of PFOS in acetonitrile with NaCl and RVC electrodes. Chemical shifts are referenced to potassium acetate at 181.2 ppm.

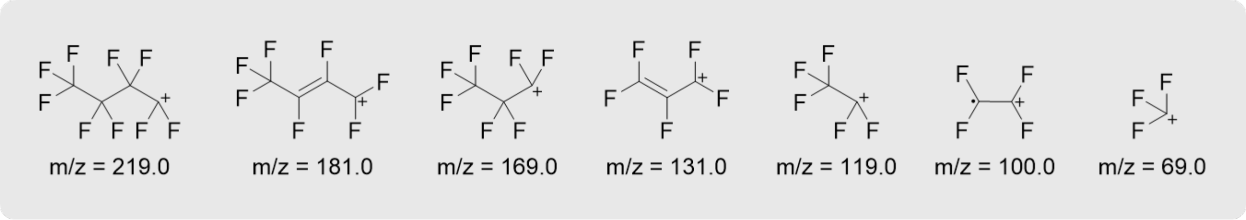

Figure S14. Proposed short-chain organofluorine species produced in GC-MS from the volatile byproducts of the electrochemical degradation of PFOS.

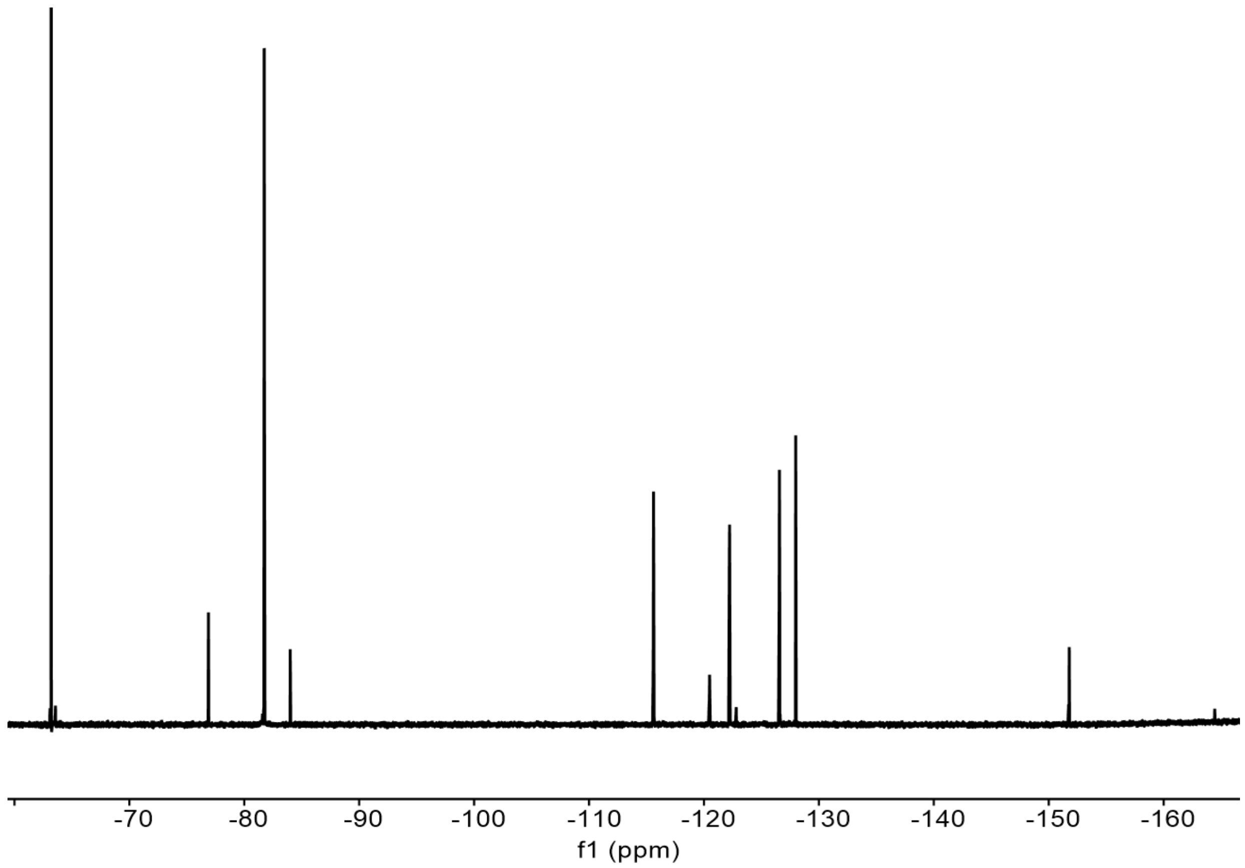

Figure S15. ^{19}F NMR spectrum (DMSO-d_6) of reaction mixture after electrolysis of PFHxS in acetonitrile with NaCl and Pt electrodes. Chemical shifts are referenced to α,α,α-trifluorotoluene at -63.2 ppm.

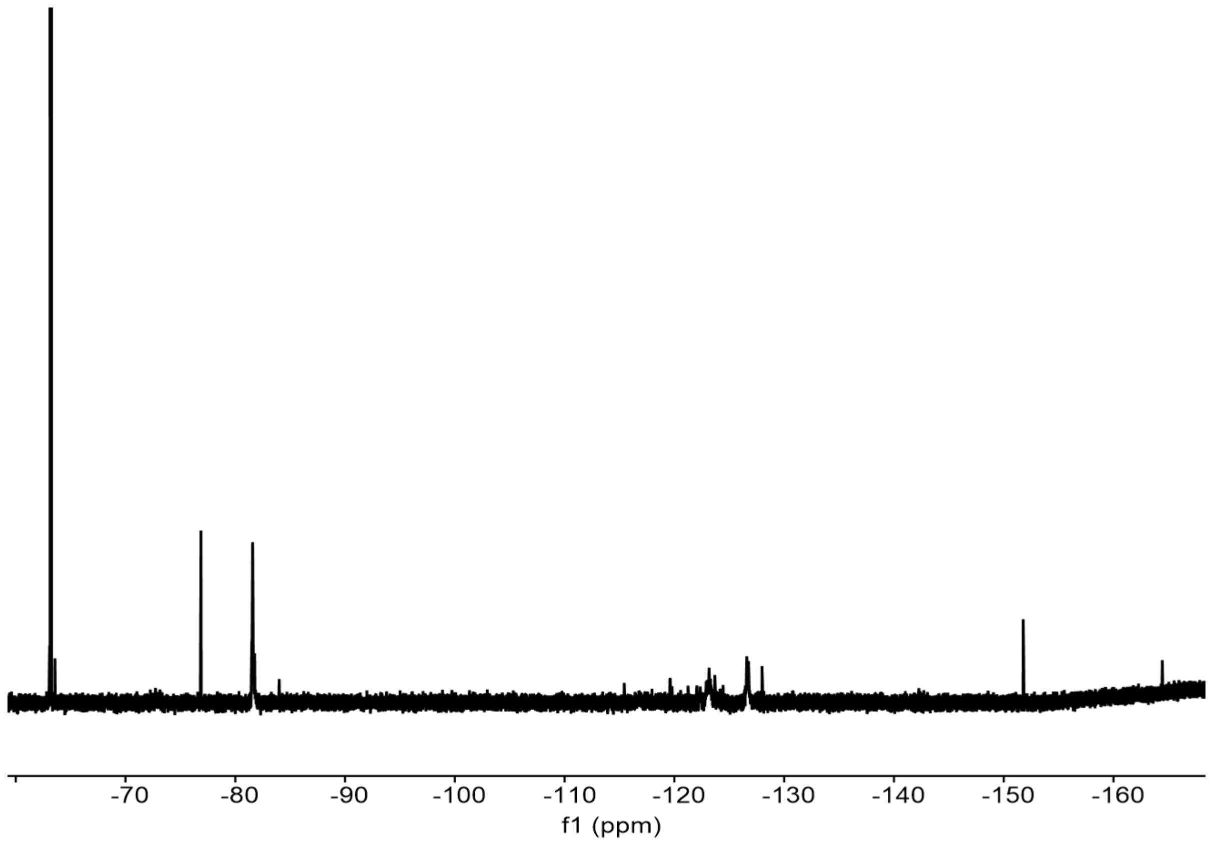

Figure S16. ^{19}F NMR spectrum (DMSO-d_6) of reaction mixture after electrolysis of PFBS in acetonitrile with NaCl and Pt electrodes. Chemical shifts are referenced to α,α,α-trifluorotoluene at -63.2 ppm.

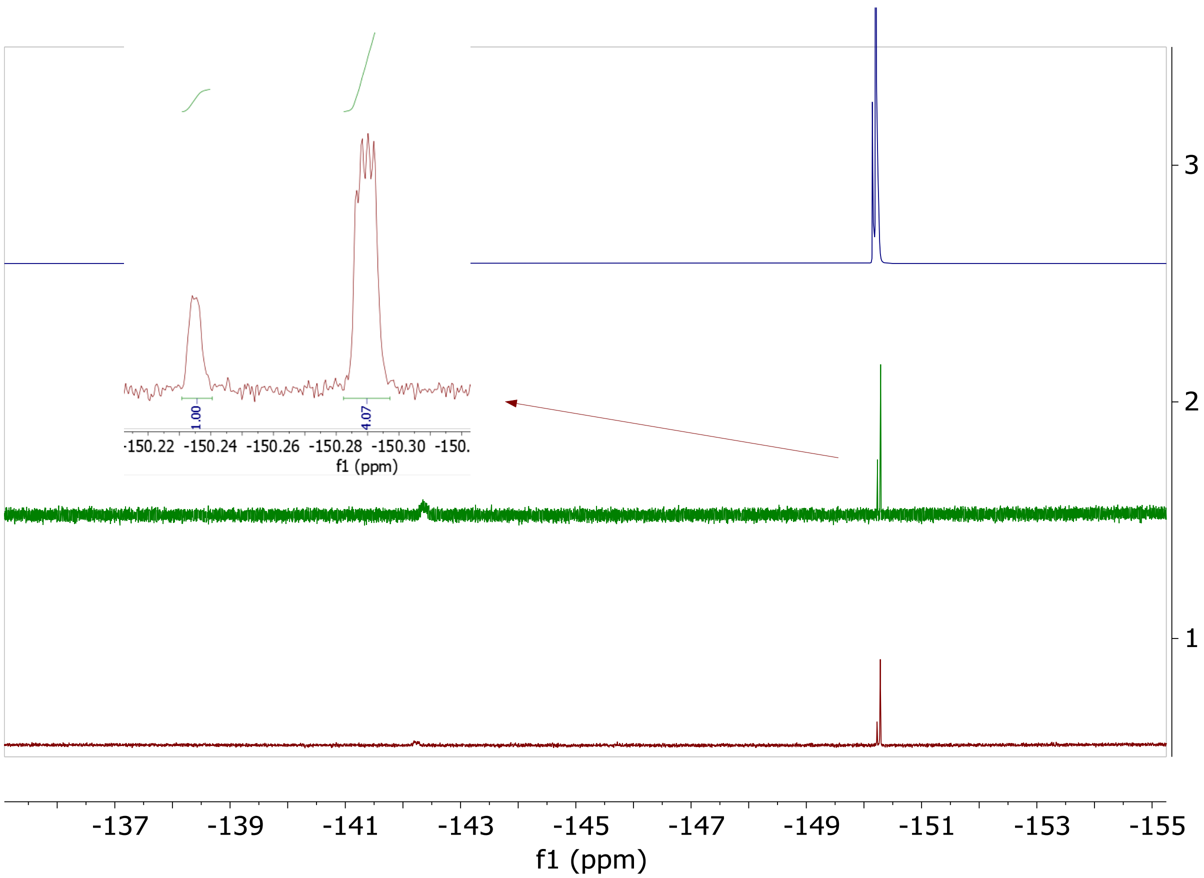

Figure S17. ^{19}F NMR spectra highlighting presence of the tetrafluoroborate anion post electrolysis. Top: a pure sample of $NaBF_4$ in DMSO-d_6. Middle: spectrum (DMSO-d_6) of reaction mixture after electrolysis of PFOS in acetonitrile with NaCl and Pt electrodes in a polyethylene vial. Bottom: spectrum from the standard electrolysis conditions in a glass vial. The insert of the zoomed middle spectrum shows the expected splitting pattern and integration for the tetrafluoroborate anion. Chemical shifts are referenced to α,α,α-trifluorotoluene at -63.2 ppm. For the sample from the reaction in the polyethylene vial, BF_4^- could only have stemmed from a reaction between HF and the NMR tube glass. Since this reaction between the borosilicate glass and HF readily occurs during sample preparation, we can assume that it occurs during the typical reaction in a glass vial.

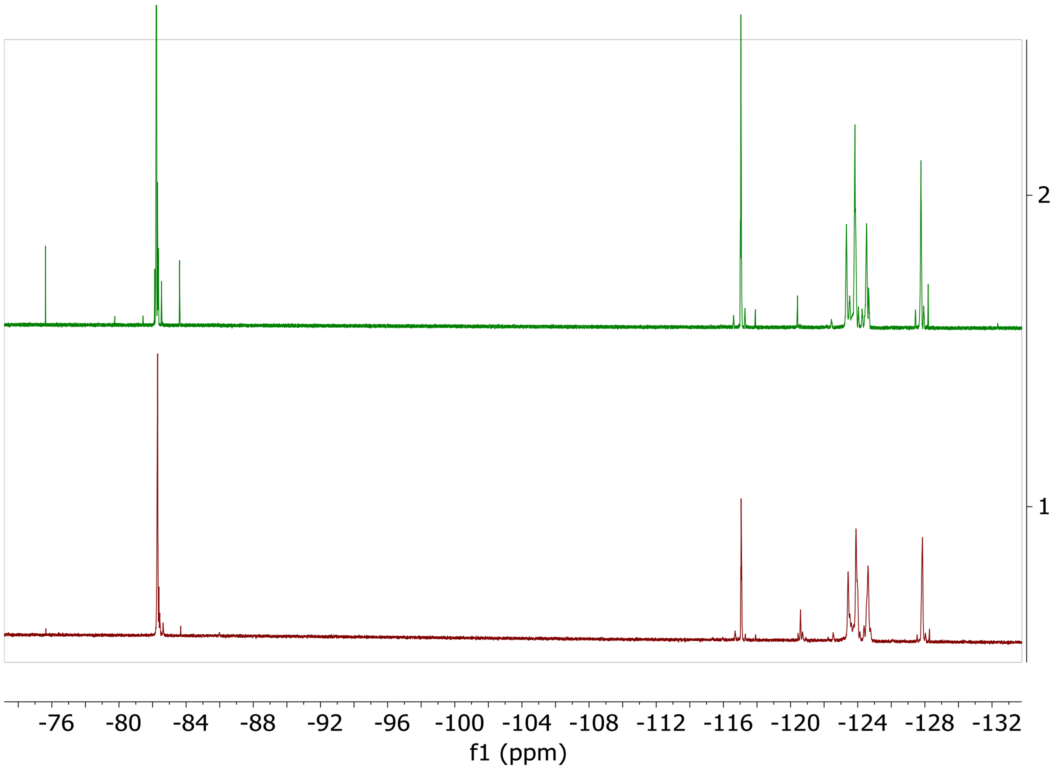

Figure S18. ^{19}F NMR spectra (DMSO-d_6) of electrolysis products when the reaction is performed in a polyethylene vial (top) vs a glass vial (bottom). The major product peaks align between the two reactions, suggesting the prominent peaks in the spectra are from PFAS degradation. Chemical shifts are referenced to α,α,α-trifluorotoluene at -63.2 ppm.

References

[41] J. H. Shin, G. Parkin, "Fluoro Complexes of Permethyltantalocene, Cp*2TaF3 and [Cp*2TaF2][BF4]: Facile Formation of a Tetrafluoroborate Complex via Corrosion of Borosilicate Glass" *Organometallics* **1998**, *17*, 5689–5696.

[42] M. G. Freire, C. M. S. S. Neves, I. M. Marrucho, J. A. P. Coutinho, A. M. Fernandes, "Hydrolysis of Tetrafluoroborate and Hexafluorophosphate Counter Ions in Imidazolium-Based Ionic Liquids" *J. Phys. Chem. A* **2010**, *114*, 3744–3749.

[43] A. Serva, N. Dubouis, A. Grimaud, M. Salanne, "Confining Water in Ionic and Organic Solvents to Tune Its Adsorption and Reactivity at Electrified Interfaces" *Acc. Chem. Res.* **2021**, *54*, 1034–1042.

[44] M. L. Pegis, J. A. S. Roberts, D. J. Wasylenko, E. A. Mader, A. M. Appel, J. M. Mayer, "Standard Reduction Potentials for Oxygen and Carbon Dioxide Couples in Acetonitrile and N,N-Dimethylformamide" *Inorg. Chem.* **2015**, *54*, 11883–11888.

[45] L. Wang, J. Lu, L. Li, Y. Wang, Q. Huang, "Effects of chloride on electrochemical degradation of perfluorooctanesulfonate by Magnéli phase Ti4O7 and boron doped diamond anodes" *Water Research* **2020**, *170*, 115254.
